# The Effect of Storage Conditions on the Properties of Sourdough Starters, Mature Sourdoughs and Quality of the Bread

**DOI:** 10.3390/foods15213835

**Published:** 2026-10-27

**Authors:** Mateusz Gertchen, Anna Czubaszek, Mikołaj Wosik, Alan Gasiński, Witold Pietrzak, Joanna Kawa-Rygielska

**Affiliations:** Department of Fermentation and Cereals Technology, Wrocław Univeristy of Environmental and Life Sciences, Chełmońskiego 37, 51-630 Wrocław, Poland; mateusz.gertchen@upwr.edu.pl (M.G.); alan.gasinski@upwr.edu.pl (A.G.); witold.pietrzak@upwr.edu.pl (W.P.); joanna.kawa-rygielska@upwr.edu.pl (J.K.-R.)

**Keywords:** sourdough, sourdough starter, storage, quality, rye bread

## Abstract

Sourdough production is an important technological process in bakeries. An increasing number of artisan bakeries choose to store their sourdough starters under refrigerated conditions. This study aimed to evaluate changes in the properties of bakery sourdoughs produced from rye sourdough starters stored at 4 °C for 48, 96, and 144 h, and to determine the effect of sourdough starter storage on the quality of the resulting bread. Refrigerated storage of sourdough starter for 48, 96 and 144 h affected the pH and acidity of the sourdough starters but did not influence these parameters in the mature sourdoughs. The concentrations of fermentation products, such as acetic acid, ethanol, glycerol, and lactic acid, changed during storage, and it was found that ethanol increased with longer storage time in sourdough starters. Refrigerated storage did not affect key bread quality characteristics, including crumb moisture, bread yield, baking loss, or loaf volume. Sensory evaluation showed that bread made from a starter stored for 144 h was the least acceptable in terms of taste. Overall, these findings establish a clear operational guideline, demonstrating that rye sourdough starters can be safely stored under refrigerated conditions for up to 96 h without compromising bread quality.

## 1. Introduction

Fermented products have been produced since ancient times. Even then, the use of a mixture of flour and water to produce sourdough through spontaneous fermentation was widely known. Sourdough fermentation is used to improve the texture, flavor, and shelf life of bread [1,2]. Various types of flour can be used to produce sourdough. In Eastern Europe and Scandinavia, rye and barley flours are the typical raw materials for sourdough production. In contrast, in South America and the Mediterranean countries (Italy, France), sourdoughs are produced from wheat flour. Rye flour is characterized by a lower content of gluten proteins than wheat flour. These proteins are responsible for forming the appropriate structure of wheat dough. The structure of rye doughs is determined by the amount and properties of starch and pentosans, which prevent the hydration of gluten proteins. For this reason, acidifying part of the dough is necessary to create the appropriate structure of rye doughs. The sourdough fermentation process reduces dough viscosity and converts insoluble pentosans into soluble ones, which can form a pentosan network [3]. Important factors affecting the quality and properties of sourdough include fermentation temperature and the yields of individual phases. By controlling the physical parameters of sourdough, it is possible to direct the fermentation process and shape its properties. Maintaining sourdough at higher temperatures (30–40 °C) results in higher acidity. As the ambient temperature increases, the amount of lactic acid, synthesized by lactic acid bacteria present in the sourdough, also increases. At the same time the synthesis of acetic acid and aromatic compounds decreases. A similar relationship was observed when changing the yield of the phases. Sourdoughs with a yield above 200 are characterized by a higher lactic acid content and a lower amount of acetic acid, aromatic compounds, and carbon dioxide [4]. Sourdoughs display a high species diversity among lactic acid bacteria. At the beginning of spontaneous fermentation, the environment is quickly dominated by homofermentative and heterofermentative lactic acid rods, as well as cocci (Pediococci). The most frequently isolated bacterial representatives are *Lactobacillus casei*, *Lactobacillus rossiae*, *Lactobacillus ponis*, *Fructilactobacillus sanfranciscensis*, and *Leuconostoc* species [5]. The organisms that subsequently proliferate in sourdoughs are yeasts, with the main representatives being *Candida humilis*, *Saccharomyces exiguus*, *Saccharomyces cerevisiae*, and *Torulaspora* species. Initially, bacteria use the growth and nutrient substances present in the young sourdough to synthesize lactic and acetic acid, which raise the acidity of the environment and create favorable developmental conditions for yeasts. These microorganisms, using simple sugars, amino acids, vitamins, and trace elements, carry out ethanolic fermentation, during which, in addition to ethanol and carbon dioxide, they synthesize aromatic compounds such as aldehydes, ketones, and alcohols. These metabolites are responsible for the characteristic aroma of sourdoughs and the bread baked from them [6].

It is worth noting that sourdough bread has a lower glycemic index than bread made with industrial yeast. The lactic acid produced during fermentation slows down the digestion of starch present in the bread, while acetic acid prolongs the time food spends in the stomach [7,8].

Sourdoughs also affect the rheological and organoleptic properties of doughs, as well as the quality of bread. In rye doughs, lowering the environmental pH increases the swelling capacity of pentosans. Acidification allows more gas to be retained in rye dough, resulting in the structure characteristic of rye bread. In the production of wheat bread, adding sourdough is optional and serves to introduce subtle acidity and increase the bread’s microbiological stability. After sourdough is introduced into wheat dough, changes occur in its viscoelastic properties [7]. These changes result from increased proteolytic enzyme activity, which is triggered by lower environmental pH [9]. A positive effect of sourdough addition on the quality and yield of wheat bread has been demonstrated [7].

Although refrigerated storage of sourdough starters is a common practice in modern artisanal and industrial bakeries—particularly to manage production schedules over weekends or extended operational breaks—there is a significant lack of systematic scientific data regarding the precise biochemical limits of such storage. Specifically, the threshold at which prolonged cold storage (up to several days) begins to irreversibly alter starter microflora activity, metabolite profiles, and ultimately the sensory quality of the final rye bread remains poorly understood. Therefore, the aim of this study was to comprehensively evaluate changes in the physicochemical properties and metabolite profiles of rye sourdough starters stored under refrigerated conditions (4 °C) for up to 144 h, as well as to determine the critical time thresholds affecting mature sourdough behavior and the technological and sensory quality of the resulting bread.

## 2. Materials and Methods

### 2.1. Research Material

Rye flour (type 720) obtained from Młyn Ciechanowiec Jachimczuk i Stanisławski Sp. z o.o. was used in the study. Sourdough starter used in this study was provided by artisanal bakery.

### 2.2. Sourdough Starters Production

Sourdoughs starters were prepared using the three-stage method, as described by Brandt and Gänzle [10]. An starter culture to direct the fermentation process and accelerate acidification was used sourdough mature from an artisanal bakery. After preparing the first sourdough, three starters were taken from it and subsequently stored at 4 °C (typical sourdough refrigeration temperature used in the bakeries) for 48, 96, and 144 h (samples accordingly SS48, SS96, SS144). Control sample was sourdough starter, which was not stored (SS0).

### 2.3. Mature Sourdough and Bread Production

Mature sourdoughs were prepared from the stored sourdough starters using the three-stage method [10]. Mature sourdoughs are described as follows: MS0—mature sourdough made from non-stored starter, MS48—mature sourdough made from starter stored for 48 h, MS96—mature sourdough made from starter stored for 96 h, MS144—mature sourdough made from starter stored for 144 h.

Rye bread was produced from the mature sourdoughs according to the recipe given in Table 1. The parameters of sourdough fermentation and bread production were the same for all variants. The dough was mixed in a farinograph mixer (Brabender GmbH, Duisburg, Germany). The production method allowed for the production of four different breads: RB0—rye bread made from sourdough obtained from non-stored starter, RB48—rye bread made from sourdough obtained from starter stored for 48 h, RB96—rye bread made from sourdough obtained from starter stored for 96 h, RB144—rye bread made from sourdough obtained from starter stored for 144 h.

### 2.4. Analytical Methods

#### 2.4.1. Flour Quality Assessment

The quality of rye flour was assessed using the following analyses: determination of moisture content by the drying method according to PN-A-74108:1996 [11], determination of the rheological properties of flour pastes using a amylograph (Brabender GmbH, Duisburg, Germany) according to PN-EN ISO 7973:2016-01 [12].

#### 2.4.2. Assessment of Sourdough Acidity

The pH of the sourdough was assessed using an Apera Instruments PH600S-Z (Shanghai, China) meter and titrable acidity was measured using method of Brandt and Gänzle [10].

#### 2.4.3. Determination of Acetic Acid, Ethanol, Lactic Acid and Glycerol in Sourdoughs

Concentration of acetic acid, ethanol, lactic acid and glycerol was measured using high performance liquid chromatography (HPLC). A portion of the sourdough starter was liquefied in 50–60 mL of 0.2% NaOH, and shaken for one hour. The solution was then quantitatively transferred into a 100 cm^3^ volumetric flask using distilled water. One ml of Carrez I and one mL of Carrez II solutions were added, mixed, and the distilled water was added to acquire total volume of 100 cm^3^. Fifty cm^3^ of the solution was then transferred into 50 cm^3^ Falcon tubes and centrifuged for 15 min at 5000 rpm. The supernatant was collected from above the sediment and filtered through fluted filter paper. One milliliter of the sample was transferred to chromatographic vial and analysed using LC-20AD chromatograph (Shimadzu, Kyoto, Japan) with and refractive index detector (RID-10A), based on Buksa [13] and Kawa-Rygielska & Pietrzak [14] methods.

#### 2.4.4. Analysis of the Bread Quality

The baked loaves were weighed both hot and after cooling in order to determine baking loss and bread yield. After cooling, the bread volume was measured using a SAWY apparatus (Sadkiewicz Instruments, Bydgoszcz, Poland), and the results were expressed as bread volume per 100 g of flour and as specific volume (volume per 1 g of bread). The following parameters were also analyzed: crumb porosity, according to the Mohs scale [15] and bread moisture content, according to PN-A-74108:1996 [11].

#### 2.4.5. Analysis of the Volatiles

Volatiles were measured in sourdough, mature sourdough and bread using headspace solid-phase microextraction and gas chromatography coupled with mass spectrometry (HS-SPME-GC-MS). At first, the volatiles had to be adsorbed onto toe SPME fiber. Samples (0.5 g of sourdoughs or 1 g of the bread crumb) were transferred into the 20 mL headspace vial, followed by the addition of five mL of saturated NaCl solution (at 20 °C). twenty microliters of the internal standard (2-undecanone in cyclohexane, concentration: 1 µg/1 mL) were added, followed by the addition of 1 cm magnetic stirrer. Vial was closed with a cap equipped with a PTFE septum and placed on a heatplate of a magnetic stirrer, set at 25 °C and 200 rpm. SPME needle was inserted through the septum into the vial. After 5 min of equilibration, SPME fiber (DVB/CAR/PDMS, 50/30 µm) was exposed to the sample headspace and volatiles were adsorbed for 40 min. After adsorption, fiber was injected into the GC-2010 (Shimadzu, Kyoto, Japan) inlet set at 195 °C in a splitless mode (2 min hold time). Oven temperature program as follows: 40 °C, hold 1 min, increase at 8 °C per min to 195 °C, hold 1 min. The column used was 30 m × 0.25 mm × 0.2 µm DB-5 ms column. Column flow was set at 1.1 mL/min (using constant linear velocity) and the carrier gas used was helium. Detection of the compounds was performed using GC-MSQP 2010 SE (Shimadzu, Kyoto, Japan) in scan mode (*m*/*z* 35–400) using electron ionization, with the interface temperature at 195 °C and mass spectrometer at 250 °C. Compounds were identified based on the similarity search in NIST 17 library (components with less than 90% similarity search were removed), as well as their linear retention indexes. Concentration of the identified components was determined semi-quantitatively, assuming response factor of 1 relative to the internal standard area.

#### 2.4.6. Sensory Evaluation of the Baked Bread

A sensory evaluation was also carried out using a 9-point hedonic scale, where: 1—dislike extremely; 2—dislike very much; 3—dislike moderately; 4—dislike slightly; 5—neither dislike nor like; 6—like slightly; 7—like moderately; 8—like very much; 9—like extremely (the study was approved by the Research Ethics Committee of the Wrocław University of Environmental and Life Sciences, resolution No. NON00000.0020.1.10.3.2025, 14 July 2025).

The following quality attributes were evaluated by sensory methods: appearance, crust color, crumb color, texture, aroma, and taste. A total of 20 people participated in the bread evaluation. Each panelists had a previous experience in the sensory evaluation of the bread and was regular consumer of bread. Samples were coded with random three digit numbers. Halves of loaves were presented for the crust color and crumb color. Each panelist was given round crumb cutouts (3 cm diameter, 1 cm thickness) for the assessment of aroma, texture and taste. Bread was served at ambient (20 °C) temperature. Panelists were asked to clean the palate using unsalted crackers and water after each sample.

### 2.5. Data Processing

Acquired results are presented as means ± standard deviation. One-way analysis of variance was performed for the analysis of sourdough starters, mature sourdoughs and bread, utilizing Duncan test for determination of the homogenous groups using Statistica 13.0 Software (Statsoft, Kraków, Poland). Two principal component analyses (PCA) were conducted to compare the assessed properties of sourdough starters and mature sourdoughs; as well as breads. The single-linkage agglomerative method was used, employing the Euclidean measure to determine the distance between each alkaloid content and steeping degree and temperature.

## 3. Results and Discussion

Selecting an appropriate raw material is essential for producing high-quality sourdough. The most important component influencing the physicochemical properties and quality of sourdough is flour [16]. When considering the technological suitability of flour, particular attention should be paid to its moisture content, which determines flowability and regulates the rate of enzymatic transformations and microbiological activity. According to the PN-A-74032:2002 standard [17], the moisture content of rye flour should not exceed 15%. The rye flour examined had a moisture content of 10.9% (Table 2), which is consistent with current standards.

The quality of rye flour is determined by numerous parameters that establish its baking value. Among these, the properties of the starch-amylolytic complex are of particular importance, which is why the assessment of rye flour primarily focuses on evaluating this complex [16]. One of the instruments used to assess this complex is the Brabender amylograph, which allows for an indirect evaluation of the baking value of rye flour. For rye flours used in the production of sourdough acids and bread, the optimal maximum viscosity of the flour paste should fall within the range of 400–600 AU [18]. The final gelatinization temperature of rye flour with good baking properties should not exceed 68 °C and not be lower than 55 °C [19]. Stępniewska et al. [16] and Sobczyk et al. [18] found that the use of rye flour with a maximum flour paste viscosity exceeding 700 AU slows down the acidification process of sourdough acids, and bread obtained from such flour has a small volume. In the examined rye flour, the gelatinization process began at 57.5 °C and ended at 79.5 °C (Table 2). The maximum viscosity of the flour paste achieved 780 AU. According to Stępniewska et al. [20], a final gelatinization temperature above 68 °C indicates low susceptibility of starch to enzymatic activity, which can lead to bread defects such as a crumbling or cracked crumb.

Using analyzed rye flour and an artisanal bakery sourdough starter, sourdough was produced by the three-phase method. However, it has to be pointed out, that the used rye flour was characterized with rather low enzymatic activity, therefore, results acquired in this study might not be transferrable to every type of rye product. Sourdough starters were taken from the mature sourdough and subsequently stored at 4 °C for 48, 96, and 144 h. During storage, biochemical transformations occur in the starters. The rate and direction of these transformations are influenced by the quality of the flour as well as the microflora of the environment and of the rye flour itself [10,21]. The maturity of the sourdough is determined mainly by its pH, which in a sourdough of appropriate quality should fall within the range of 4.3–3.9. During fermentation, the degree of acidity (titratable acidity) can also be measured; however, one should not rely solely on this parameter, as it is less precise than pH measurement. Based on the results of both parameters, it is possible to assess whether the sourdough is sufficiently mature. Still, achieving an appropriate pH is a key condition for obtaining good properties [10]. Subsequent discussion about the changes in the microbial community are only based on the inference of metabolites and lack direct microbiological evidence. Many factors, such as fermentation time, temperature, and the type of flour used, influence changes in pH and acidity. Yeasts and lactic acid bacteria residing in the starters, as a result of metabolizing the carbohydrates present in the flour, produce metabolites that affect the pH and degree of acidity of the starters and the mature sourdoughs. The microorganisms present in the starters are mainly mesophiles or thermophiles, whereas lactic acid bacteria and yeasts whose growth optima fall within the temperature range prevailing during storage under refrigerated conditions (4–14 °C) occur considerably less frequently [2,10,22].

The application of reduced storage temperature for the sourdough starters or for conducting the sourdoughs falling within the range of 4–15 °C, can slow down the fermentation process. Although the activity of microorganisms should be considerably lower than at their optimal growth temperatures, they nevertheless continue to carry out processes that lower the environment’s pH.

**Figure 1:**
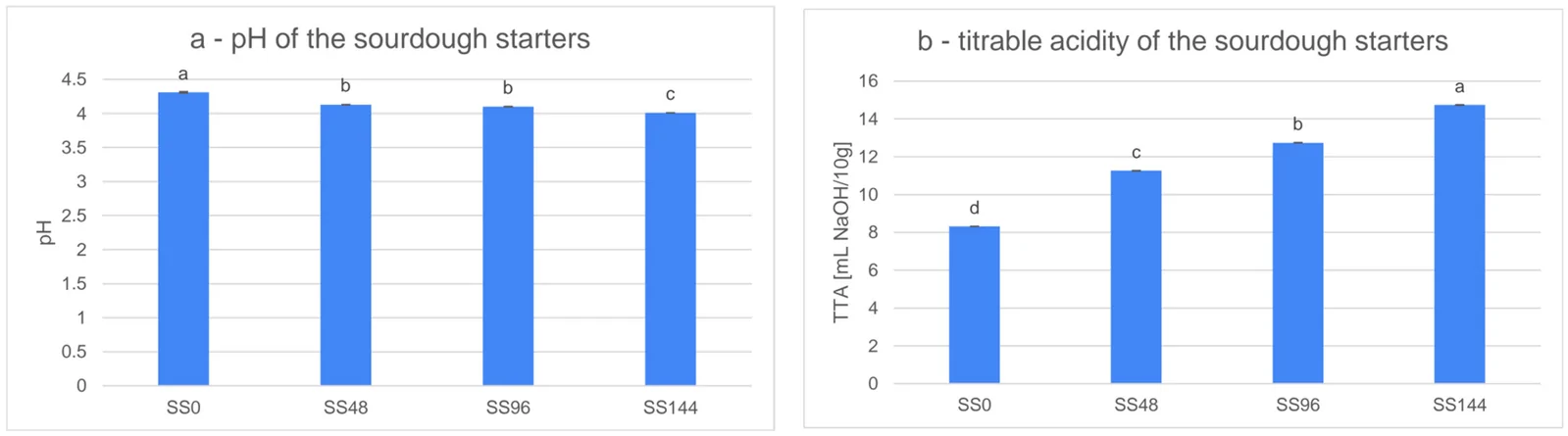
Value of pH (**a**) and total titratable acidity (**b**) of sourdough starters. Abbreviations: SS0—non-stored sourdough starter, SS48—sourdough starter stored for 48 h, SS96—sourdough starter stored for 96 h, SS144—sourdough starter stored for 144 h. Different letters in columns indicate different homogenous groups at *p* = 0.05 according to Duncan’s test. The data are expressed as means (*n* = 3).

As reported by Brandt and Gänzle [10], a starter taken immediately after the production of sourdough has a pH of 3.9–4.3. The starter in our study was characterized by a pH of 4.31 and an acidity of 8.32 (Figure 1a,b). Storage of the starters under refrigerated conditions decreased pH, with the lowest value observed for the starter stored for 144 h at 4 °C (Figure 1a). The degree of acidity (TTA) of the starters stored under refrigerated conditions increased from 8.32 (non-stored starter) to 11.26 after 48 h, 12.74 after 96 h, and 14.74 after 144 h of storage (Figure 1b).

Generally, the metabolism of the microorganisms present in the starters slows at 4 °C but is not inhibited. Therefore, extending the fermentation time under low storage temperatures of the starters can lead to changes in their acidity. The greatest decrease in pH and TTA value was observed between the non-stored sourdough starter (SS0) and the starter stored for 48 h (SS48). In the subsequent hours of storage, no further changes of this magnitude in these parameters were observed. The increase in the degree of acidity (TTA) and the decrease in pH possibly result from the activity of lactic acid bacteria and yeasts.

**Figure 2:**
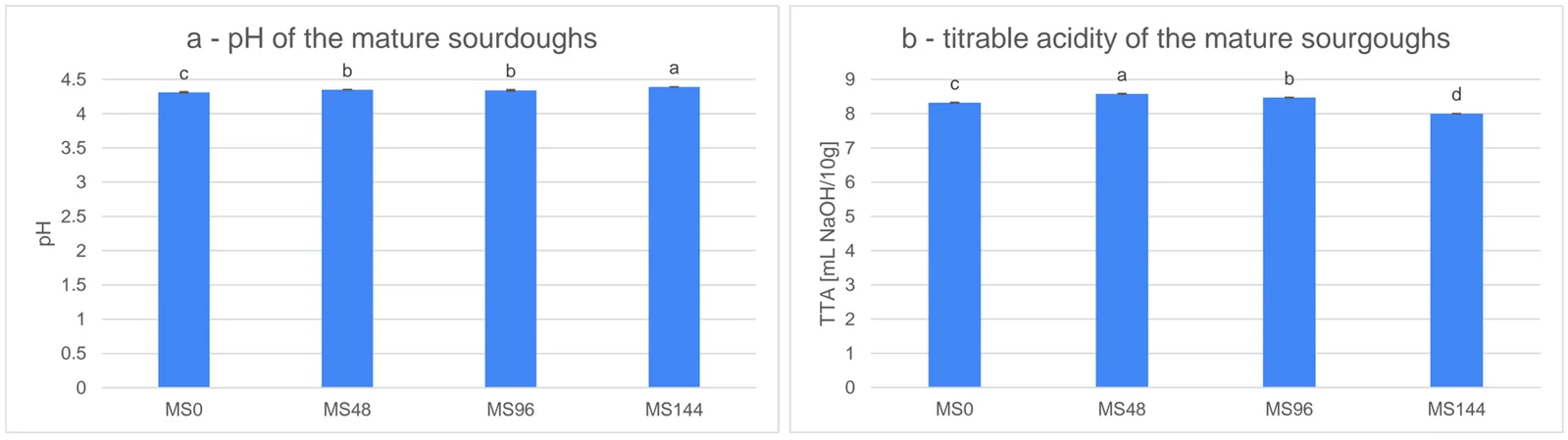
Value of pH (**a**) and total titratable acidity (**b**) of mature sourdoughs. Abbreviations: MS0—mature sourdough made from non-stored starter, MS48—mature sourdough made from starter stored for 48 h, MS96—mature sourdough made from starter stored for 96 h, MS144—mature sourdough made from starter stored for 144 h. Different letters in columns indicate different homogenous groups at *p* = 0.05 according to Duncan’s test. The data are expressed as means (*n* = 3).

The sourdoughs produced from starters stored under refrigerated conditions were characterized by a slightly higher pH value than 4.3, which Brandt and Gänzle [10] suggest as the optimal value (4.35 after 48 h, 4.34 after 96 h, and 4.39 after 144 h of storage) (Figure 2a). During storage of the starters, a tendency toward decreasing pH and increasing TTA was observed, whereas the opposite trend was found for the sourdoughs—with storage, pH tended to increase, and TTA tended to decrease. However, the pH and TTA values obtained for the sourdoughs produced by starters stored for different periods were similar and they were not technologically meaningful (Figure 2a,b).

The reduction in environmental pH and the increase in acidity result from the accumulation of fermentation products in the environment, such as lactic acid, acetic acid, bacteriocins, aldehydes, and ethanol [10,23,24]. Changes in the concentrations of yeast and lactic acid bacteria metabolites in mature sourdoughs and starters may result from their degree of acidification. According to Yue et al. [24], pH values oscillating within the range of 3.8–4.7 favor the accumulation of metabolites produced by yeasts (ethanol, glycerol, esters) and lactic acid bacteria (organic acids, ethanol, esters, acetates. Extending the fermentation process of sourdough starters and sourdoughs increases the concentrations of metabolites responsible for the acidification of rye doughs.

**Figure 3:**
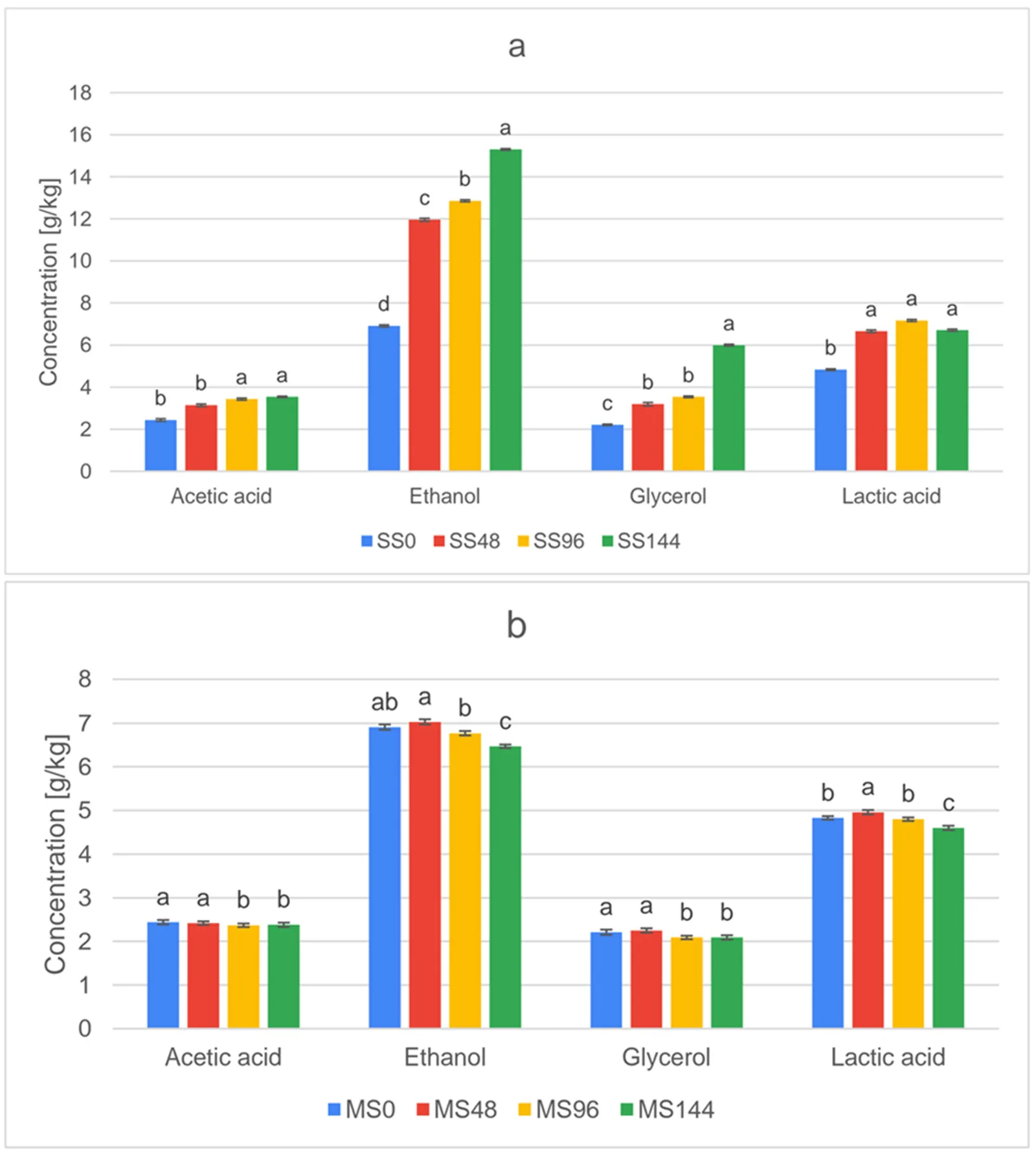
Concentration of fermentation products in sourdough starters (**a**) and mature sourdoughs (**b**) Abbreviations: SS0—non-stored sourdough starter, SS48—sourdough starter stored for 48 h, SS96—sourdough starter stored for 96 h, SS144—sourdough starter stored for 144 h, MS0—mature sourdough made from non-stored starter, MS48—mature sourdough made form starter stored for 48 h, MS96—mature sourdough made form starter stored for 96 h, MS144—mature sourdough made form starter stored for 144 h. Different letters in columns for a given compound indicate different homogenous groups at *p* = 0.05 according to Duncan’s test. The data are expressed as means (*n* = 3).

In the present study, the effect of time on the concentration of fermentation products in starters and sourdoughs was characterized (Figure 3a,b). It has been statistically shown that refrigerated storage of starters increases the concentration of ethanol and acetic acid. The lowest concentration of ethanol (6.91 g/kg) and acetic acid (2.44 g/kg) was observed in the non-stored starter (Figure 3a) while the highest concentration of ethanol (1.52 g/kg) was observed after 144 h of storage the starter. In the case of acetic acid, the highest concentrations were observed after 96 (3.43 g/kg) and 144 (3.54 g/kg) hours of storage (Figure 3a). No significant differences were found among the tested starters in lactic acid and glycerol concentration. Nevertheless, it was noted that the sourdough starter contained 1.5 times more lactic acid after 96 h of storage than the non-stored starter.

On the basis of the concentration of analysed substances, it could be hypothesized that lowering the ambient temperature to refrigerated conditions slowed down the metabolism of microorganisms. Extending the fermentation time at low temperatures may compensate for the unfavorable effect of low temperature (4 °C). For this reason, the concentration of ethanol and acetic acid increased with the extended storage time of the starters at 4 °C (Figure 3a).

As the concentration of fermentation products increases, their toxicity toward the microorganisms inhabiting the starters also increases [10,22]. Adverse effects of fermentation products and inappropriate environmental temperatures affect the activity of microorganisms and may alter the amounts of metabolites produced by lactic acid bacteria and yeasts. Brandt and Gänzle [10] and Yue et al. [24] point to the influence of organic acid and ethanol concentrations on the activity of lactic acid bacteria and yeasts. In their studies, they demonstrate that a reduction of pH value below 4.5, caused by the accumulation of organic acids in the environment, reduces or halts (at pH below 4.0) the metabolic activity of microorganisms such as *Fructilactobacillus sanfranciscensis*, *Torulaspora holmii*, and *Candida humilis*. The slowdown in acetic acid production in starters stored for 96 and 144 h may indicate the cessation of lactic acid bacteria metabolism. Metabolites could be produced as a result of lactic acid bacteria activity, such as bacteriocins, H_2_O_2_, and acids, as well as yeast metabolites: ethanol, higher alcohols, and esters, could act antagonistically toward the microorganisms present in sourdoughs and starters.

Refreshing the sourdough by adding fresh water and flour to the starter reduces the amount of products produced during fermentation. At the same time, the addition of a fresh portion of flour supplies microorganisms residing in the sourdough with growth substances, and can stimulate their metabolism, and enable the continuation of further life processes [2,10,23].

In the present study, it was observed that the sourdoughs contained lower levels of the analyzed compounds than the starters from which they were produced. Analysis of variance revealed significant differences in the levels of lactic and acetic acids, ethanol, and glycerol among the sourdoughs tested. The highest concentration of lactic acid (0.50 g/kg) and ethanol (0.71 g/kg) was observed in the sourdough produced from a starter stored under refrigerated conditions for 48 h (Figure 3b). The lowest concentrations of ethanol (6.47 g/kg) and lactic acid (4.60 g/kg) were observed in sourdoughs produced from starters stored under refrigerated conditions for 144 h. The glycerol and acetic acid content was significantly higher in sourdoughs prepared with starters SS 0 and SS 48 than in those prepared with starters SS 96 and SS 144. These statistically significant differences are of no technological significance (Figure 3b).

The differences in lactic acid and ethanol concentrations may result from reduced microflora activity in the starter, which serves as the specific inoculum for the sourdough. High concentrations of ethanol (15.30 g/kg) and acetic acid (3.54 g/kg) in the starter may have inhibited certain microorganisms (e.g., *Candida humilis*, *Fructilactobacillus sanfranciscensis*, *Candida holmii*) present in the starter, thereby affecting the sourdough fermentation process. The low pH (4.01) of the starter stored under refrigerated conditions for 144 h may also have had an inhibitory effect on part of the microflora, as reflected in the amount of products obtained during fermentation. Yue et al. [24] report that fermentation products accumulated in the environment inhibit the metabolic activity of, among others, *Fructilactobacillus sanfranciscensis*, a strain most frequently isolated from mature sourdoughs.

Using sourdough in bread production not only shapes its aroma and flavor but also affects the bread’s quality characteristics: volume, crumb moisture, crumb hardness, and shelf life. Sourdough-produced bread is not as well leavened as that made with baker’s yeast [10,25]. To obtain a better-leavened sourdough bread, the addition of baker’s yeast is often required. The amount of yeast in naturally fermented (spontaneous fermentation) sourdoughs is insufficient [25,26,27].

According to Banu et al. [21], the moisture content of sourdough bread ranges from 45.62 to 46.52 [%]. The moisture content obtained in the present study differs only slightly from the data reported by these authors. The volume of bread per 100 g of flour ranged from 336.67 (RB 48) to 362.0 cm^3^/100 g flour (RB 96), while the specific volume ranged from 2.46 to 2.67 [cm^3^/1 g bread] (Table 3).

The specific volume, volume per 100 g of flour, crumb moisture content as, well as bread yield and baking loss of bread made from starters stored under refrigerated conditions for 48, 96, and 144 h did not deteriorate compared to bread made from starters not subjected to refrigerated storage. Bread volume is influenced by the yeast present in the starter and in the mature sourdough. During prolonged fermentation, the primary energy source (carbohydrates) in the medium (mature sourdough or starter) can become depleted, while compounds toxic to yeast cells (alcohol, organic acids, H_2_O_2_) accumulate, resulting in the gradual death of yeast cells [2,6,10]. The refrigerated temperature (4 °C) at which the starters were stored reduced the enzymatic activity of the flour and the metabolic activity of the microflora. This may have limited the negative effects of fermentation, which lasted up to 144 h. This is likely why refrigerated storage of the starters did not negatively affect the volume per 100 g of flour or the specific volume of the rye bread. The porosity of bread made from starters stored under refrigerated conditions was variable 6 (RB 144) to 8 points (RB 48). Among the bread samples, the one with the finest air bubbles evenly distributed throughout the crumb structure was the bread made from starter stored under refrigerated conditions for 144 h (6 points). Photographs of the baked bread crumbs are provided as Supplementary Material (Figure S1). The remaining samples had less evenly distributed air bubbles in the crumb and contained more irregularly sized air bubbles.

**Figure 4:**
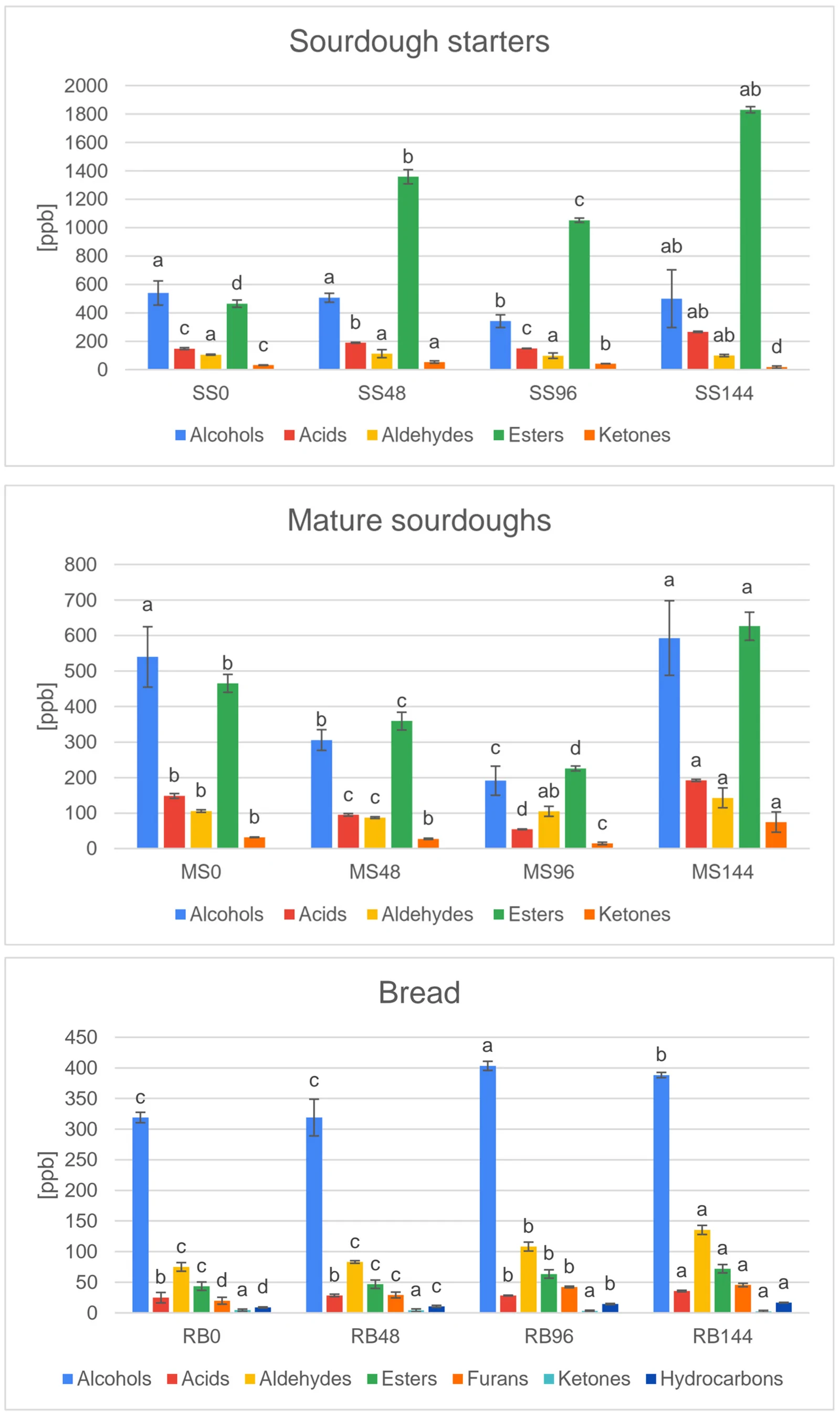
Concentration of volatile compounds from different chemical classes of volatiles in sourdough starters, mature sourdoughs and bread. Abbreviations: SS—sourdough starter, MS—mature sourdough, RB—bread, 0, 48, 96, 144—the storage time of sourdough starter. Different letters in columns for a given compound indicate different homogenous groups at *p* = 0.05 according to Duncan’s test. The data are expressed as means (*n* = 3).

To evaluate the effect volatile dynamics, VOC data were aggregated into major functional chemical classes: alcohols, esters, aldehydes, ketones, acids, furans, and hydrocarbons. Data is presented in the Figure 4.

Alcohols and esters constituted the primary volatile classes across all sourdough treatments. In the sourdough starters, volatile accumulation exhibited a distinct non-linear pattern during storage. Rather than a continuous rise, stored starters experienced a sharp initial surge in total alcohols and esters at 48 h of storage (SS48; alcohols: 1526.88 ± 31.45 ppb, esters: 499.18 ± 50.05 ppb) relative to baseline (SS0; alcohols: 839.90 ± 85.11 ppb, esters: 288.54 ± 25.35 ppb). This initial expansion was followed by a temporary reduction at 96 h of storage (SS96; alcohols: 1142.78 ± 44.74 ppb, esters: 375.19 ± 15.08 ppb), before resuming accumulation to reach maximum storage values at 144 h (SS144; alcohols: 1921.72 ± 203.22 ppb, esters: 638.58 ± 21.66 ppb). This non-linear accumulation in the starters can hypothetically reflect ongoing microbial metabolism, cell lysis, and organic acid/alcohol accumulation during extended storage [28].

Interestingly, when stored starters were refreshed into mature sourdoughs via the 3-stage fermentation method, the concentrations of total alcohols and esters dropped significantly compared to their corresponding stored starter baselines. For instance, mature sourdough prepared from 96-h stored starter (MS96) contained only 333.51 (±41.05) ppb total alcohols and 124.09 (±6.94) ppb total esters, compared to 1142.78 (±44.74) ppb and 375.19 (±15.08) ppb in the unrefreshed SS96 starter.

This pronounced decrease is primarily driven by the progressive dilution of the accumulated volatile matrix when mixing the stored starter with fresh flour and water across three sequential propagation stages. Furthermore, the introduction of fresh substrate shifts microbial metabolic activity back toward exponential biomass growth and primary organic acid synthesis rather than secondary volatile accumulation. Minor functional classes (aldehydes, acids, and ketones) exhibited far lower baseline fluctuations throughout both storage and mature sourdough preparation, demonstrating that the 3-stage refreshment process predominantly impacts major alcohol and ester pools [29,30].

When mature sourdoughs were incorporated into bread formulations and baked, the broad volatile distribution underwent a clear secondary transformation. Alcohols remained the primary functional class in the final bread matrix (ranging from 318.98 ± 8.47 ppb in RB0 to 403.28 ± 7.47 ppb in RB96), confirming that volatile alcohols carry over effectively into the bread crumb.

However, thermal energy penetrating the crumb during baking initiated secondary thermal degradation pathways. Most notably, furans emerged as a major functional group in the final bread (19.77 ± 1.76 ppb in RB0, rising to 45.42 ± 5.48 ppb in RB144), alongside measurable concentrations of hydrocarbons (peaking at 16.22 ± 0.54 ppb in RB 144). Total aldehydes also expanded markedly compared to the sourdough matrices, reaching 135.42 (±7.44) ppb in RB144. These shifts illustrate a mechanistic distinction across the production chain: while starter storage and 3-stage refreshment establish the baseline alcohol and ester profile, thermal reactions during baking (Maillard reactions and lipid oxidation) govern the generation of crust-derived furans and aldehydes [31,32].

The advantages of sourdough bread include its flavor, crumb structure, and aroma. In the present study, the baked loaves of rye bread were subjected to organoleptic evaluation, with each bread sample assessed by 20 individuals. The following parameters were evaluated: appearance, crust color, crumb color, texture, aroma, and taste. The assessment was conducted using a 9-point hedonic scale, in which 1 (dislike extremely) was the lowest value and 9 (like extremely) was the highest. Scores for all attributes ranged from 7.07 (texture RB144) to 8.27 (appearance RB0), indicating high consumer acceptance of all breads tested (Table 4).

In a study conducted by Xu et al. [33], lowering the fermentation temperature from 20–30 °C to 10 °C improved the following characteristics: taste, aroma, appearance, and overall quality of the bread. Troadec et al. [34] report that extending fermentation time also improves bread taste and aroma. In our own studies, using a starter stored at 4 °C for 48, 96, and 144 h for bread production, there were no significant differences in appearance (7.73–8.27), crust color (8.00–8.20), crumb color (7.47–8.00), texture (7.47–7.93), or aroma (7.27–7.93). Analysis of variance showed significant variation only in taste. According to the panelists, the best taste (8.07) was found in bread produced from the non-stored starter, while the significantly least acceptable taste (7.13) was found in bread produced from the starter stored under refrigerated conditions for 144 h. Bread made from starters stored under refrigerated conditions for 48 (7.73) and 96 (7.67) hours was less acceptable than bread made from the non-stored starter, but more acceptable than bread made from the starter stored for 144 h (Table 4).

This assessment may have been influenced by metabolites from sourdough microflora activity, which the panelists perceived unfavorably. Microorganisms in sourdough produce organic acids, alcohols, aldehydes, and ketones that affect bread quality. Extending fermentation time increases the accumulation of these compounds in sourdough, leading to changes in bread quality [10,22,33]. Perhaps the fact, that the bread made from starter stored for 144 h contained highest concentration of acids, aldehydes and ketones might results in worse perception of this sample, as excessive concentration of various volatiles from those groups might results in panelists sensing unpleasant off-flavours.

**Figure 5:**
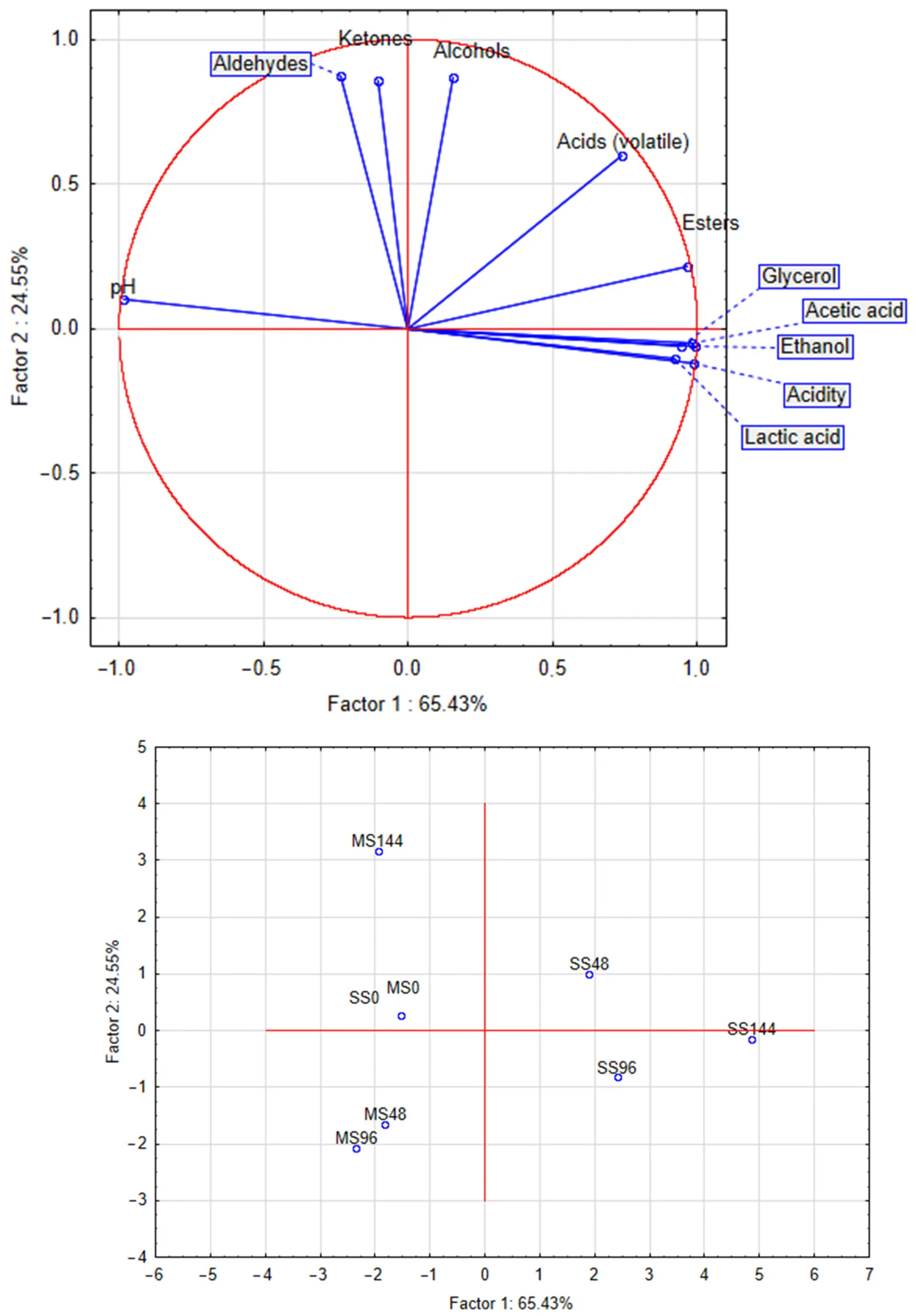
Charts of the PCA analysis of the sourdough starters and mature sourdoughs.

**Figure 6:**
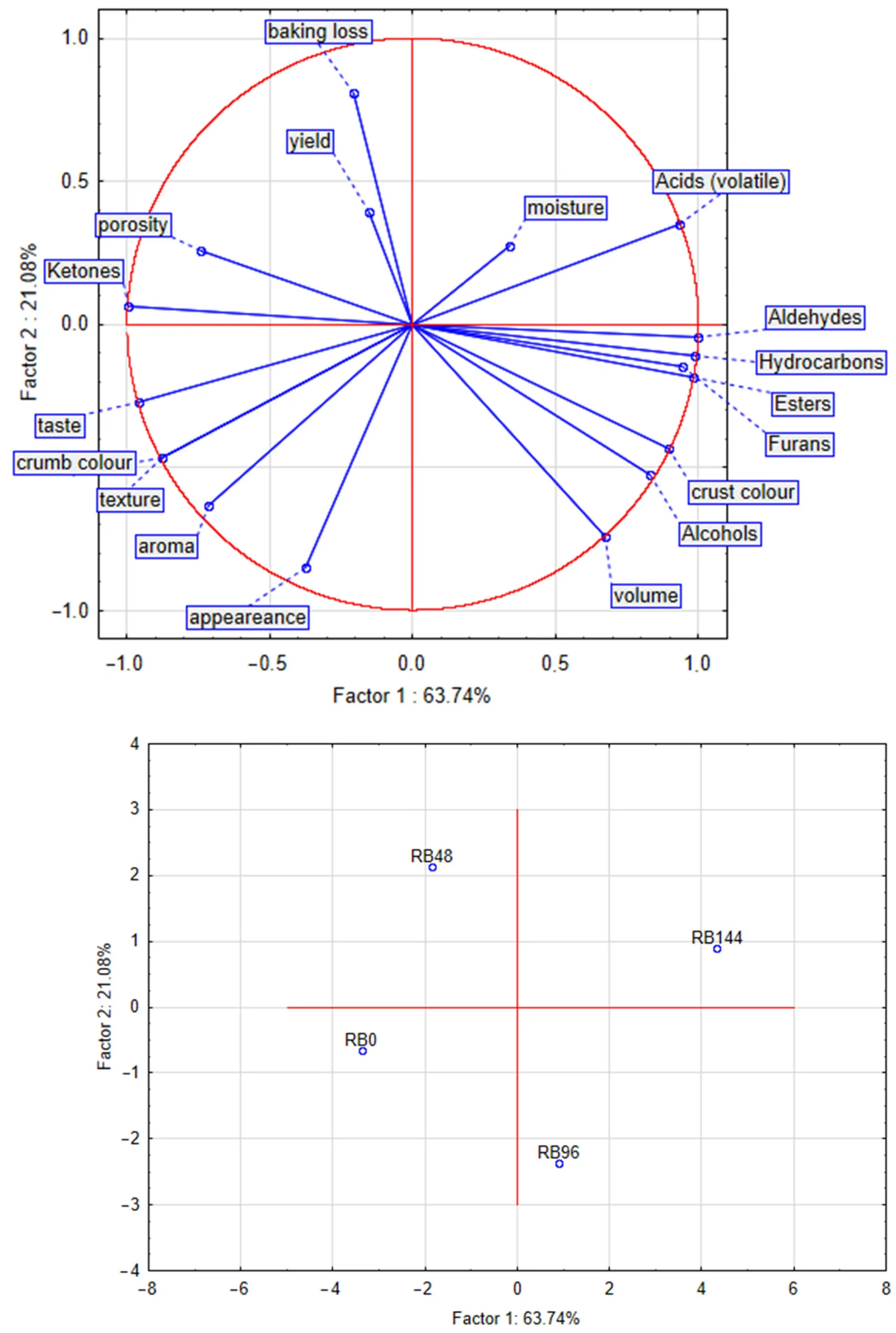
Charts of the PCA analysis of the bread.

On the data acquired in this study, principal component analysis (PCA) was performed. Analyses clearly separated both the sourdough matrices (PCA 1) and the resulting bread samples (PCA 2). Results are presented on Figure 5 and Figure 6.

For the sourdough starters and mature sourdoughs, first and second principal component (PC1 and PC2) accounted for 89.98% of the total variance (65.43% and 24.55%, respectively). PC1 mainly represented the primary fermentation progress, showing a strong inverse relationship between pH and organic acids, ethanol, and titratable acidity. Storing the starter for longer periods (SS48 to SS144) caused a heavy buildup of lactic acid, acetic acid, ethanol, and glycerol. However, the subsequent refreshment step to produce mature sourdoughs pushed the samples back along PC1, effectively balancing out the main acidity parameters. The exception was MS144 (from the 144-h stored starter), which separated distinctly on PC2 due to notably higher levels of volatile secondary metabolites like alcohols, ketones, and aldehydes.

These differences in the starters directly influenced the final bread quality (PCA 2), explaining 84.82% of total variance: PC1 63.74%, PC2 21.08%). Breads made with fresh or moderately stored starters (RB0 and RB48) plotted on the negative side of PC1. This region correlated with higher sensory scores for appearance, taste, aroma, crumb color, and porosity. On the other hand, bread made from the 144-h starter (RB144) shifted far to the right on PC1. It showed a high accumulation of volatile compounds—mainly esters, furans, aldehydes, and volatile acids—but received lower sensory ratings. Meanwhile, the 96-h sample (RB96) was mainly associated with larger loaf volume, darker crust color, and specific alcohols.

Overall, while extended starter storage builds up a high acid load, the refreshment stage buffers this acidity while keeping specific volatile precursors intact. Storage time can therefore be used to control the end product. Intermediate storage (48–96 h) gives the best balance for loaf volume, yield, and consumer sensory acceptance, whereas 144-h storage shifts the bread toward an intensely aromatic profile at the expense of traditional texture and taste.

From a practical perspective, these findings address a critical gap in modern bakery operations concerning extended cold storage schedules (such as weekend holds or operational downtime). While previous studies have primarily examined standard fermentation parameters or short-term maintenance, our results provide a systematic multi-factorial profile of starter aging up to 144 h. A key and novel observation of this study is the remarkable resilience of physical bread characteristics: crumb moisture, specific volume, baking loss, and yield remained unaffected even when utilizing starters stored for up to 144 h. This indicates that the structural matrix of rye dough possesses a high buffering capacity against variations in starter activity. However, the critical tipping point lies in the sensory profile. Prolonged storage beyond 96 h, specifically at 144 h, led to a marked decrease in taste acceptability. This divergence demonstrates that while physical leavening and structure can be maintained, the metabolic shifts and accumulation of fermentation products (such as elevated acetic acid and ethanol) in the cold-stressed starter exert a direct, negative influence on the final organoleptic properties. Consequently, this study establishes that while technical failure does not occur immediately, 96 h represents a critical operational threshold for maintaining optimal sensory quality in commercial rye bread production.

## 4. Conclusions

In conclusion, this study demonstrates that refrigerated storage (4 °C) of rye sourdough starters for up to 144 h significantly modifies starter acidity and metabolite accumulation (notably acetic acid and ethanol), yet does not adversely affect the primary physical quality parameters (moisture, volume, baking loss, and yield) of the resulting rye bread. Nevertheless, extended cold storage introduces a distinct sensory threshold: starters stored for 144 h resulted in bread with reduced taste acceptability due to excessive metabolite accumulation. From an industrial and artisanal standpoint, these findings establish a clear, data-backed operational guideline: rye sourdough starters can be safely maintained under refrigerated conditions for up to 96 h without compromising either technological performance or sensory quality. This work provides a valuable practical benchmark for optimizing bakery production schedules and managing starter stability during extended operational breaks.

## Figures and Tables

**Table 1:** Quantitative composition of raw materials used for the preparation of rye breads.

Ingredients	Quantity
Sourdough	200 g
Rye flour	200 g
Water	to obtain a dough consistency of 300 BU (113.5 ± 3 mL)
Salt	5.4 g

**Table 2:** Characteristics of rye flour (type 720).

Parameter	Unit	Mean Value
Moisture content	[%]	10.9 ± 0.2
Maximum viscosity of flour paste	[AU]	780 ± 10
Initial gelatinization temperature	[°C]	57.5 ± 0.2
Final gelatinization temperature	[°C]	79.5 ± 0.5

**Table 3:** Quality characteristics of rye bread.

Type of Bread	Moisture of Crumb [%]	Volume of Bread per 100 g of Flour [cm^3^/100 g Flour]	Specific Volume [cm^3^/1 g Bread]	Bread Yield [%]	Baking Loss [%]	Crumb Porosity in Mohs Scale [Points]
RB0	46.07± 0.46 ^a^	344.7 ± 2.5 ^ab^	2.49 ± 0.11 ^ab^	138.2 ± 4.1 ^a^	9.47 ± 0.59 ^a^	7 ± 0.5 ^ab^
RB48	45.67 ± 0.59 ^a^	336.7 ± 3.1 ^b^	2.46 ± 0.07 ^b^	136.7 ± 2.2 ^a^	10.19 ± 0.63 ^a^	8 ± 0.3 ^a^
RB96	45.56 ± 0.41 ^a^	362.0 ± 2.9 ^a^	2.67 ± 0.09 ^a^	135.5 ± 3.0 ^a^	9.50 ± 0.42 ^a^	7 ± 0.0 ^ab^
RB144	46.27 ± 0.72 ^a^	355.3 ± 2.6 ^a^	2.58 ± 0.05 ^a^	137.7 ± 2.8 ^a^	9.61 ± 0.31 ^a^	6 ± 0.0 ^b^

Abbreviations: RB0—rye bread made from sourdough obtained from non-stored starter, RB48—rye bread made from sourdough obtained from starter stored for 48 h, RB96—rye bread made from sourdough obtained from starter stored for 96 h, RB144—rye bread made from sourdough obtained from starter stored for 144 h. Different letters in columns indicate different homogenous groups at *p* = 0.05 according to Duncan’s test. The data are expressed as means (*n* = 3).

**Table 4:** Organoleptic evaluation of bread.

Type of Bread	Appearance	Crust Color	Crumb Color	Texture	Aroma	Taste
RB0	8.27± 0.92 ^a^	8.00 ± 0.02 ^a^	8.00 ± 0.58 ^a^	7.93 ± 0.58 ^a^	7.87 ± 0.58 ^a^	8.07 ± 0.92 ^a^
RB48	7.60 ± 1.12 ^a^	8.00 ± 0.50 ^a^	7.53 ± 0.45 ^a^	7.47 ± 0.45 ^a^	7.67 ± 1.00 ^a^	7.73 ± 0.63 ^ab^
RB96	8.13 ± 0.83 ^a^	8.20 ± 0.36 ^a^	7.80 ± 0.36 ^a^	7.60 ± 0.36 ^a^	7.93 ± 0.45 ^a^	7.67 ± 0.78 ^ab^
RB144	7.73 ± 1.00 ^a^	8.20 ± 0.63 ^a^	7.47 ± 0.58 ^a^	7.07 ± 0.58 ^a^	7.27 ± 0.83 ^a^	7.13 ± 0.83 ^b^

Abbreviations: RB0—rye bread made from sourdough obtained from non-stored starter, RB48—rye bread made from sourdough obtained from starter stored for 48 h, RB96—rye bread made from sourdough obtained from starter stored for 96 h, RB144—rye bread made from sourdough obtained from starter stored for 144 h. Different letters in columns indicate different homogenous groups at *p* = 0.05 according to Duncan’s test. The data are expressed as means (*n* = 3).

## Data Availability

The original contributions presented in this study are included in the article/Supplementary Material. Further inquiries can be directed to the corresponding author.

## Supplementary Materials

The following supporting information can be downloaded at: https://www.mdpi.com/article/10.3390/foods15213835/s1, Figure S1: Photographs of the baked bread crumbs.
