# Supplementary material for: The Effect of Storage Conditions on the Properties of Sourdough Starters, Mature Sourdoughs and Quality of the Bread
Source: Foods. 2026 Oct 27;15(21):3835. doi: 10.3390/foods15213835 (PMC13762471; doi:10.3390/foods15213835)
Supplement: Supplementary file 1 [file foods-15-03835-s001.zip › Figure S1.pdf]

Sample Crumb of bread

RB 0

RB 48

RB 96

RB 144

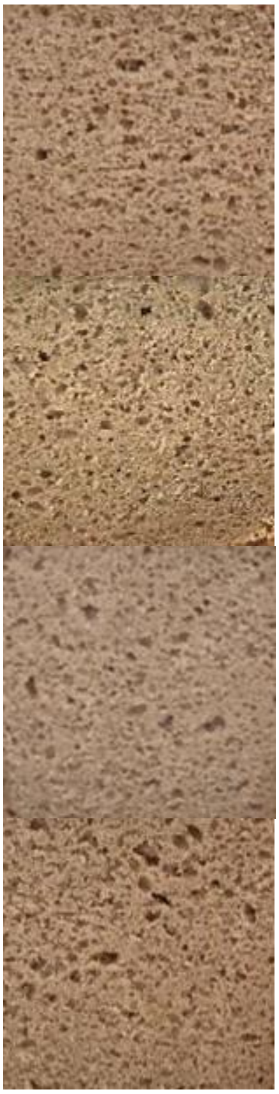

Figure S1. Photographs of the baked bread crumbs
